# Quantitative Analyses Reveal Novel Roles for *N-*Glycosylation in a Major Enteric Bacterial Pathogen

**DOI:** 10.1128/mBio.00297-19

**Published:** 2019-04-23

**Authors:** Sherif Abouelhadid, Simon J. North, Paul Hitchen, Prerna Vohra, Cosmin Chintoan-Uta, Mark Stevens, Anne Dell, Jon Cuccui, Brendan W. Wren

**Affiliations:** a Department of Pathogen Biology, London School of Hygiene and Tropical Medicine, London, United Kingdom; b Department of Life Sciences, Imperial College London, London, United Kingdom; c The Roslin Institute and Royal (Dick) School of Veterinary Studies, University of Edinburgh, Edinburgh, United Kingdom; The Sanger Institute

**Keywords:** glycosylation, host-microbe interaction, microbial physiology, pathogenesis, proteomics

## Abstract

In eukaryotes, glycosylation plays a role in proteome stability, protein quality control, and modulating protein function; however, similar studies in bacteria are lacking. Here, we investigate the roles of general protein glycosylation systems in bacteria using the enteropathogen Campylobacter jejuni as a well-defined example. By using a quantitative proteomic strategy, we were able to monitor changes in the C. jejuni proteome when glycosylation is disrupted. We demonstrate that in C. jejuni*, N-*glycosylation is essential to maintain proteome stability and protein quality control. These findings guided us to investigate the role of *N-*glycosylation in modulating bacterial cellular activities. In glycosylation-deficient C. jejuni, the multidrug efflux pump and electron transport pathways were significantly impaired. We demonstrate that *in vivo*, fully glycosylation-deficient C. jejuni bacteria were unable to colonize its natural avian host. These results provide the first evidence of a link between proteome stability and complex functions via a bacterial general glycosylation system.

## Abstract (precis)

Advances in genomics and mass spectrometry have revealed several types of glycosylation systems in bacteria. However, why bacterial proteins are modified remains poorly defined. Here, we investigated the role of general *N-*linked glycosylation in a major food poisoning bacterium, Campylobacter jejuni. The aim of this study is to delineate the direct and indirect effects caused by disrupting this posttranslational modification. To achieve this, we employed a quantitative proteomic strategy to monitor alterations in the C. jejuni proteome. Our quantitative proteomic results linked general protein *N-*glycosylation to maintaining proteome stability. Functional analyses revealed novel roles for bacterial *N-*glycosylation in modulating multidrug efflux pump, enhancing nitrate reduction activity, and promoting host-microbe interaction. This work provides insights on the importance of general glycosylation in proteins in maintaining bacterial physiology, thus expanding our knowledge of the emergence of posttranslational modification in bacteria.

## INTRODUCTION

Glycosylation is a prevalent protein posttranslational modification found in nature. In eukaryotes, the attachment of glycans to proteins has been shown to play a central role in modulating protein folding, stability, and signaling (1). Advances in genomics and mass spectrometry have revealed several types of glycosylation systems in bacteria. Among prokaryotes, general glycosylation systems have been reported in pathogenic bacteria, including Campylobacter jejuni (2), Neisseria gonorrhoeae (3), Burkholderia cepacia (4), as well as commensal bacteria such as Bacteroides fragilis (5), indicating their abundance across the kingdom. However, the reasons why bacterial proteins are specifically modified remains poorly defined, particularly among general protein glycosylation systems.

Genetic and biochemical studies have revealed that in C. jejuni, the *N*-oligosaccharyltransferase PglB is responsible for decorating at least 50 proteins in the C. jejuni cell with the heptasaccharide GalNAc-α1,4-GalNAc-[Glcβ1,3-]GalNAc-α1,4-GalNAc-α1,4-GalNAc-α1,3-diBacNAc-β1, where GalNAc is *N-*acetylgalactosamine, Glc is glucose, and diBacNAc is 2,4-diacetamido-2,4,6-trideoxyglucopyranose. This glycostructure is linked to the asparagine residue in the acceptor sequon D/E-X_1_-N-X_2_-S/T where X_1_ and X_2_ can be any amino acid except proline (6). Comparative genomic studies have revealed the conservation of general *N-*glycosylation gene clusters across the *Campylobacter* genus (7). Mutagenesis studies demonstrated that disruption of the *N*-glycosylation pathway led to pleiotropic effects in C. jejuni. These effects include a substantial decrease in bacterial adherence and invasion capability of INT 407 intestinal epithelial cells (8), a decrease in chicken colonization (9), and a reduction in competence (10). Similar findings have also been reported in other bacteria where inactivation of their corresponding general glycosylation systems resulted in a disruption in cellular physiology and a reduction in virulence (4, 5). These combined consequences were anticipated to be due to direct or indirect disruptions of several cellular processes in glycosylation-deficient strains. However, these studies did not address what perturbations may be occurring to the bacterial cell when glycosylation is no longer possible. To answer this question, we developed a platform to investigate the role of general *N*-linked glycosylation systems in prokaryotes, using C. jejuni as a model example.

We employed tandem mass tag (TMT) isobaric 6plex labeling quantitative proteomics on wild-type and glycosylation-deficient strains. Analysis of a glycosylation-deficient strain, revealed chaperones and stress-related proteins are more abundant, indicating a central alteration in bacterial cellular activities. Thus, contrary to current understanding, the loss of glycosylation predominantly has a mass effect on the C. jejuni proteome, rather than being restricted exclusively to proteins modified by glycosylation. Phenotypically this resulted in reduction in multidrug efflux pump activity as well as impairment in nitrate reductase activity. Furthermore, we demonstrate that general *N-*glycosylation plays an important role in host-microbe interactions in the chicken colonization model. This study provides deeper insights on the role of a general *N-*glycosylation system in bacteria, demonstrating that protein posttranslational modification is critical to a plethora of bacterial cellular activities.

## RESULTS

### *N-*glycosylation is essential for proteome stability.

**FIG 1:**
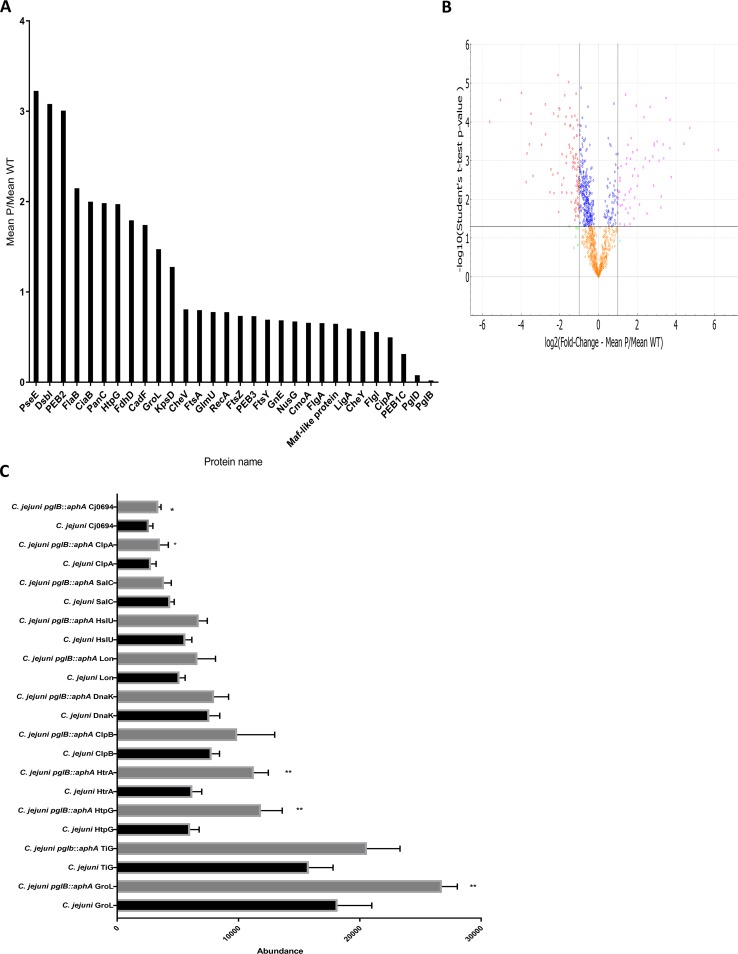
Quantitative comparison of wild-type C. jejuni and C. jejuni *pglB*::*aphA* proteomes. (A) Selected proteins that are statistically differentially expressed in wild-type C. jejuni and C. jejuni *pglB*::*aphA* proteomes. Proteins were analyzed by Perseus and presented according to their fold change with 95% CI. (B) Volcano plot analysis of significantly abundant proteins. The −log_10_ (Student’s *t* test) is plotted against log_2_ mean fold change of C. jejuni *pglB*::*aphA*,P C. jejuni, WT. The nonaxial horizontal line denotes *P =* 0.05, while the nonaxial vertical lines denote 0.5- and 2-fold changes, respectively. The volcano plot was generated using PANDA-view (12). (C) Differential expression of chaperones and proteases in wild-type C. jejuni and C. jejuni *pglB*::*aphA.* Data are from three biological replicates. The error bars represent standard deviations. Values that are significantly different by Student’s *t* test are indicated by asterisks as follows: *, *P* < 0.05; **, *P* < 0.01.

Quantitative proteomics serves as a tool to understand alterations in proteomes resulting from disruption of gene(s). To provide deeper insights into the role of the general glycosylation system in prokaryotes, we employed a mass spectrometry-based quantitative proteomic strategy to monitor differential protein abundance in glycosylation-deficient C. jejuni and compared this to wild-type bacteria. Proteins from both C. jejuni strains were reduced, alkylated, digested, and then labeled by tandem mass tag (TMT) 6plex isobaric labels to allow multiplex quantification for multiple samples**.** In total, 1,052 proteins out of 1,623 proteins were identified by matching peptides to the C. jejuni 11168 curated proteome. Proteins were quantitatively compared across the samples (6plex) and then filtered at 95% CI. After significance filtering, 502 proteins were found to be statistically different in the two strains; 120 proteins were more abundant and 382 proteins were less abundant in C. jejuni *pglB*::*aphA* lacking the oligosaccharyltransferase compared to the wild-type strain (Fig. 1A to C) (11, 12). Surprisingly, chaperones and proteases were found in significantly higher abundance in the glycosylation-deficient C. jejuni (Fig. 1C). Among these chaperones, we detected GroL, a periplasmic chaperone that binds to unfolded and partially folded proteins promoting folding (13), HtpG, a chaperone involved in binding to aggregated proteins and protecting the cell against environmental stresses (14), and Cj0694, a peptidyl-prolyl-*cis*/*trans* isomerase (PPIase) that interacts with the Sec translocon and promotes folding of polypeptides emerging from the translocon (15, 16). Protein degradation pathway proteins were also significantly highly abundant, notably HtrA, which is a multifunctional protein that plays a central role in proteolytically cleaving misfolded proteins as well as preventing aggregation of nonnative proteins (15, 17). This observation correlates with a significant reduction in the abundance of HspR in the same strain. HspR is a negative transcriptional regulator of chaperone-encoding genes such as *clpB, dnaK*, and *groLS*. Disruption of *hspR* enhanced thermotolerance in C. jejuni (18). Cytoplasmic chaperones and proteases such as ClpB, Lon, Trigger factor (Tig), and HslU that are reported to be involved in various arrays of protein quality mechanisms from assisting protein folding to protein degradation were also found to be differentially, but not statistically significantly, abundant in C. jejuni *pglB*::*aphA* (19) (Fig. 1C). These results indicate that disrupting *N-*linked glycosylation leads to a global alteration in the abundance of protein quality control machineries. The high abundance of chaperones and proteases suggests a potential misfolding of proteins and a rise in protein aggregation in glycosylation-deficient C. jejuni.

### Alteration in glycoprotein abundance in glycosylation-deficient C. jejuni.

Biochemical and biophysical studies of glycoproteins have revealed that *N*-linked glycans play a role in stabilizing proteins (20), promoting protein folding (21), and preventing protein aggregation (22). Enrichment analysis of the glycoproteome of C. jejuni has revealed 53 proteins to be decorated by the C. jejuni heptasaccharide GalNAc-α1,4-GalNAc-[Glcβ1,3-]GalNAc-α1,4-GalNAc-α1,4-GalNAc-α1,3-diBacNAc covalently linked to the asparagine residue in D/E-X_1_-N-X_2_-S/T where X_1_ and X_2_ can be any amino acid except proline (6, 23). Our quantitative proteomic approach managed to detect and correctly evaluate 35 glycoproteins in the samples. Out of the 35 glycoproteins detected, only 8 proteins (22.8%) were more abundant in glycosylation-deficient C. jejuni and 6 proteins (17.1%) were found to be unchanged. Interestingly, 21 proteins (60%) were found to be less abundant in C. jejuni *pglB*::*aphA.* It is unclear whether the low abundance of these proteins is due to misfolding/degradation of the proteins or is a consequence of global defects resulting from disruption of the *N-*linked glycosylation pathway. To gain more insights into the role of the C. jejuni heptasaccharide in promoting protein stability, we sought to study the functional activity of glycoproteins in C. jejuni.

### *N-*glycosylation modulates multidrug efflux pump activity.

**FIG 2:**
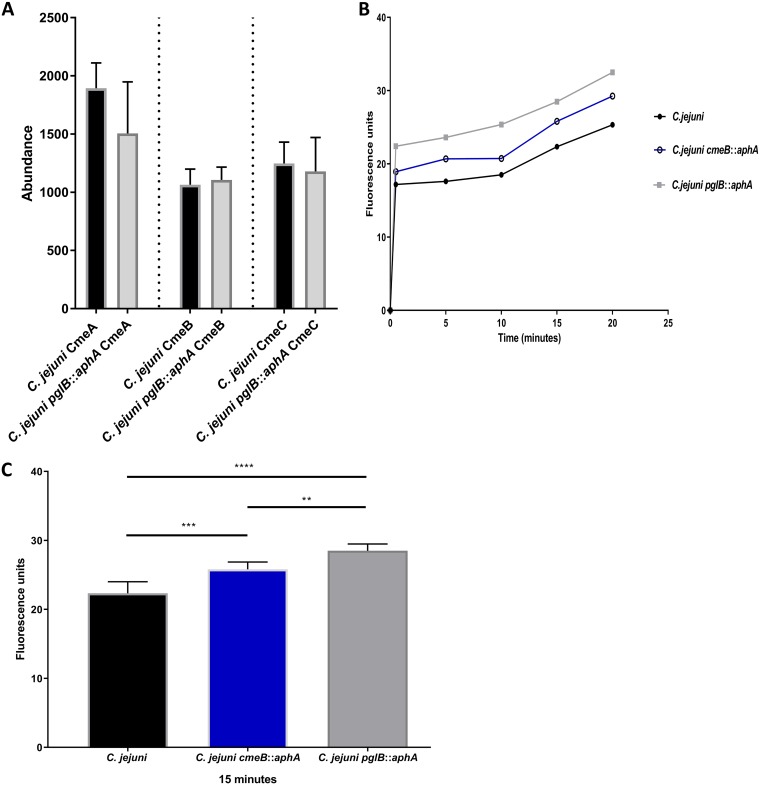
Differential expression and functional analysis of CmeABC in C. jejuni. (A) Differential expression of CmeABC in wild-type C. jejuni and C. jejuni *pglB*::*aphA.* Data are from three biological replicates, and error bars represent standard deviations. Data were analyzed by Student’s *t* test. (B) Ethidium bromide accumulation test in C. jejuni strains. Thirty milliliters of brucella broth was separately inoculated with overnight cultures of C. jejuni (black circles), C. jejuni *cmeB*::*aphA* (open circles), and C. jejuni *pglB*::*aphA* (gray squares) to an OD_600_ of 0.1. Cells were grown until an OD_600_ of 0.4 to 0.5 was reached, spun down, washed, and resuspended to an OD_600_ of 0.2 in 10 mM sodium phosphate buffer (pH 7). Cells were then incubated in a VAIN for 15 min at 37°C, and then ethidium bromide was added to a final concentration of 0.2 mg/ml. Fluorescence was read at excitation and emission for 20 min at 37°C. (B) Ethidium bromide accumulation in C. jejuni strains throughout 20 min. (C) Ethidium bromide accumulation in C. jejuni strains at 15 min. The data are means for three biological replicates, two technical replicates each, and the error bars represent standard deviations. Significance was calculated using one-way ANOVA test with multiple comparison and indicated by asterisks as follows: **, *P* < 0.01; ***, *P* < 0.001; ****, *P* < 0.0001.

A difficulty in studying the role of general glycosylation systems in bacteria is that the modified proteins often have no known function. Using a genetically tractable bacterium that modifies proteins with predictable phenotypes could help to assign more definitively the role of glycosylation. Only two protein assemblies out of the 35 detected glycoproteins in our study on C. jejuni have known phenotypes (6, 24, 25). CmeABC shares amino acid sequence homology and similar molecular architecture with other tripartite resistance-nodulation-division (RND) type multidrug efflux pumps in other Gram-negative bacteria (26). Acting as the major multidrug efflux pump in C. jejuni, CmeABC plays an important role in antibiotic resistance and host colonization (27). The tripartite RND of C. jejuni is a molecular assembly of CmeB, an inner membrane multidrug transport protein, CmeA, a periplasmic fusion protein, and CmeC, an outer membrane-associated protein channel (24), where all three complex components were found to be glycosylated (6). Our bioinformatic analysis showed the presence of two glycosylation sequons at each protein (CmeA, CmeB, and CmeC) of the CmeABC assembly. In C. jejuni *pglB*::*aphA*, CmeA was found to be 0.8-fold less abundant, while no change was seen in the levels of CmeB and CmeC compared to the wild type (Fig. 2A). To investigate the role of the heptasaccharide in multidrug resistance, we sought to assess the antibiotic resistance profile of C. jejuni and glycosylation-deficient C. jejuni. A C. jejuni *cmeB*::*aphA* mutant was constructed and showed a substantial increase in antibiotic sensitivity. In glycosylation-deficient C. jejuni, a 100% increase in sensitivity against ampicillin, erythromycin, tetracycline, and ciprofloxacin was observed compared to the wild type (see Table S1 in the supplemental material). The results may be due to a defect in CmeABC multidrug efflux leading to an accumulation of antibiotics. In order to gain further insights on the effect of glycosylation on CmeABC activity, we used the fluorescent intercalating agent ethidium bromide in an accumulation assay that enables monitoring of efflux pump activity in real time. Ethidium bromide accumulation in C. jejuni *cmeB*::*aphA* was 15% higher (*P = *0.0016) than in the wild-type strain. This result is in agreement with the antibiotic susceptibility tests. The ethidium bromide accumulation in C. jejuni *pglB*::*aphA* was 27.5% higher than in the wild type (*P < *0.0001) (Fig. 2B and C). The results show that the effect of glycosylation on C. jejuni ethidium bromide accumulation kinetics is more detrimental than inactivation of the major multidrug efflux complex. Our results demonstrate the pivotal role of the heptasaccharide in efflux activity and subsequent multidrug resistance in C. jejuni.

10.1128/mBio.00297-19.3 TABLE S1 Antimicrobial resistance of C. jejuni strains. Etest strips were used to assess minimum inhibitory concentration (MIC) of C. jejuni strains. The data represent the means from three biological replicates, with two technical replicates each. Download Table S1, TIF file, 0.1 MB. Copyright © 2019 Abouelhadid et al. 2019 Abouelhadid et al. This content is distributed under the terms of the Creative Commons Attribution 4.0 International license.

### Nitrite production impairment in glycosylation-deficient C. jejuni.

**FIG 3:**
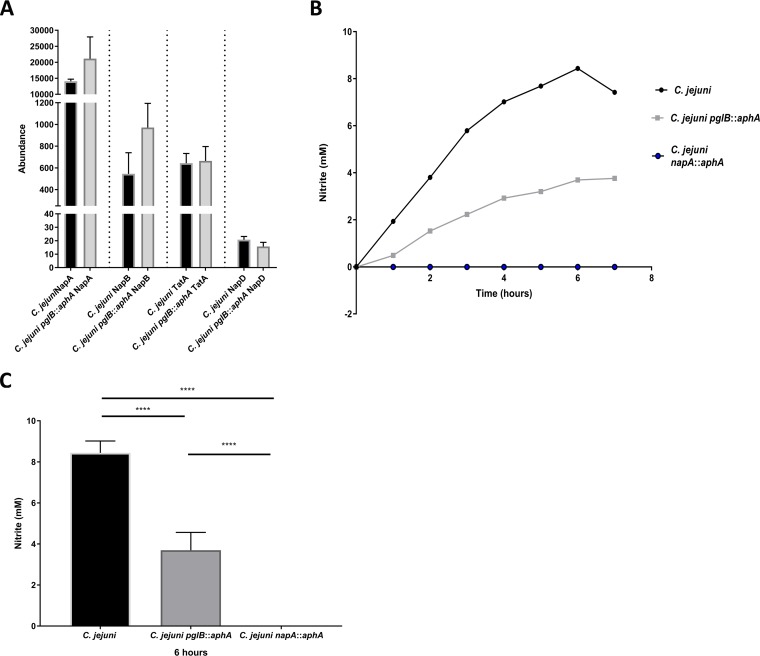
Differential expression and functional analysis of NapAB complex in C. jejuni. (A) Differential expression of NapAB, TatA, and NapD in C. jejuni and C. jejuni *pglB*::*aphA*. Data are from three biological replicates, and error bars represent standard deviations. Data were analyzed by Student’s *t* test. (B) Nitrate reduction in C. jejuni strains. Ninety milliliters of brucella broth supplemented with 200 mM nitrate was inoculated separately with wild-type C. jejuni (black circles), C. jejuni *pglB*::*aphA* (gray squares), and C. jejuni *napA*::*aphA* (blue circles) to an OD_600_ of 0.1 from an overnight culture. Cultures were incubated statically in a VAIN at 37°C. Samples were withdrawn every hour, C. jejuni cells were removed by centrifugation, and nitrate reduction was measured straight from the supernatant against nitrite standards. (B) Nitrite accumulation in the supernatant over 7 h. (C) Nitrite accumulation in the supernatant at 6 h. The data represents the mean of three biological replicates and two technical replicates, and error bars represent standard deviations. Significance was calculated using one-way ANOVA test with multiple comparison. ****, *P* < 0.0001.

To analyze whether the loss of glycosylation affects other complex assemblies in C. jejuni, we interrogated the activity of NapAB for nitrite production. NapAB is a two-subunit periplasmic nitrate reductase enzyme that is responsible for the reduction of nitrate to nitrite. NapAB consists of a twin arginine translocation substrate; NapA is a 90-kDa catalytic *bis*-molybdenum guanosine dinucleoside (MGD) cofactor binding enzyme, while NapB is a 16-kDa diheme *C*-type cytochrome glycoprotein (6, 25). Inactivation of *napA* in C. jejuni results in reduction in chicken colonization (28). Mass spectrometric analysis of the C. jejuni glycoproteome demonstrated that NapB is glycosylated at ^50^N; however, there is no experimental evidence that NapA is a glycoprotein (6). Our quantitative proteomic analysis unexpectedly revealed a 1.7 ± 0.2-fold increase in both NapA and NapB levels in C. jejuni *pglB*::*aphA* compared to the wild type (Fig. 3A). Interestingly, there was no change in abundance in twin arginine translocase (TatA) or the NapA-accompanying chaperone NapD. To discern the role of *N-*glycosylation in modulating NapAB complex activity, we sought to investigate the ability to reduce nitrate in C. jejuni and C. jejuni *pglB*::*aphA* and a newly constructed C. jejuni *napA*::*aphA* mutant. The C. jejuni strains were grown in oxygen-limited conditions for 8 h. A biphasic pattern of nitrite production was observed whereby nitrite production increases until 6 h and then starts to decline (Fig. 3B). After 1 h, a difference in nitrite production between the wild type and its glycosylation-deficient counterpart was observed, while nitrite production in the C. jejuni *napA*::*aphA* mutant could not be detected (below the limit of detection). At 6 h, C. jejuni *pglB*::*aphA* produced 63.7% (*P = *0.0018) less nitrite than the wild type did (Fig. 3C). Our results indicate a potential correlation between *N-*glycosylation and nitrate reductase activity in C. jejuni. It also suggests that despite the increase in NapAB abundance in the glycosylation-deficient strain, the activity of the NapAB complex was severely impaired compared to its glycosylated counterpart. These results indicate that *N-*glycosylation plays a role in enhancing NapAB complex efficiency in nitrite production.

### Disruption of *N-*glycosylation causes alterations in the isoprenoid biosynthetic pathway and cell morphology abnormalities.

**FIG 4:**
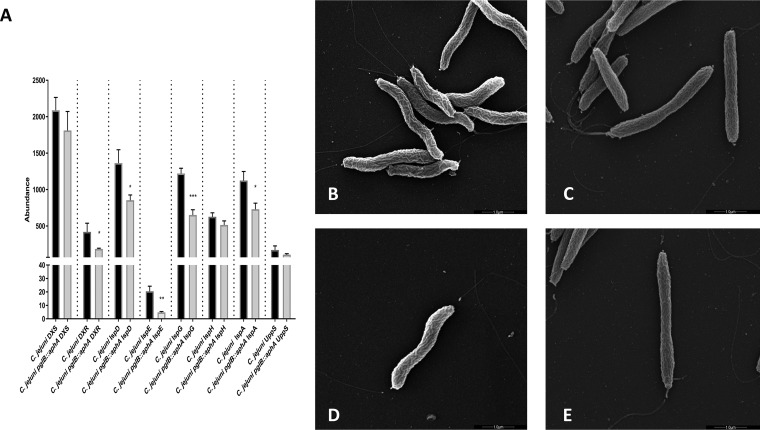
Abundance of undecaprenyl phosphate biosynthetic enzymes and cell morphology in C. jejuni strains. (A) Differential expression of MEP pathway enzymes and UppS. Data are from three biological replicates, and error bars represent standard deviations. Data were analyzed by Student’s *t* test. *, *P* < 0.05; **, *P* < 0.01; ***, *P* < 0.001. (B to E) Scanning electron micrographs of wild-type C. jejuni 11168H (B) and C. jejuni 11168H *pglB*::*aphA* (C) and of a single bacterial cell of wild-type C. jejuni 11168H (D) and a single bacterial cell of C. jejuni 11168H *pglB*::*aphA* (E). The bars represent 1.0 μm.

Isoprenoids are one of the largest naturally occurring organic compounds that are essential for maintaining bacterial cell physiology (29). They are synthesized from a five-carbon universal precursor, isopentyl diphosphate (IPP) or its isomer dimethylallyl diphosphate (DMAPP) (30). In C. jejuni, IPP is produced exclusively via 2*C-*methyl-D-erythriotol 4-phosphate (MEP) pathway that requires eight enzymatic steps. Our quantitative proteomic results show a significant reduction in MEP pathway enzymes in glycosylation-deficient C. jejuni compared to the wild-type strain (Fig. 4A). Interestingly, a 47% reduction in UppS abundance was observed in C. jejuni *pglB*::*aphA* compared to its wild-type counterpart. UppS catalyzes the sequential addition of IPP with farnesyl diphosphate to yield undecaprenyl diphosphate (Und-PP) (30). Und-P is an essential lipid carrier that plays a key role in the biosynthesis of lipooligosaccharide (LOS), capsular polysaccharide (CPS), *N-*linked glycan, and peptidoglycan in C. jejuni (31). The low abundance of UppS was accompanied by an alteration in the cell morphology of glycosylation-deficient C. jejuni, when examined via scanning electron microscopy (SEM).

C. jejuni has a helical cell morphology that along with the polar flagella, facilitates the corkscrew motility in viscous media, giving it an advantage over other bacteria (32). It has been postulated that the helical cell shape is critical for C. jejuni pathogenicity (33). Interestingly, glycosylation-deficient C. jejuni cells were shown to adapt a rod shape and are more flattened compared to the natural helical cell morphology of wild-type C. jejuni (Fig. 4D and E). We also noted a nonuniformity among the cell length in glycosylation-deficient C. jejuni that is not evident in the wild type (Fig. 4B and C). This might be due to the disruption of *N-*glycosylation in C. jejuni which also led to an alteration in Und-PP production, as shown by the reduction in isoprenoid biosynthesis and UppS, hence affecting PG biosynthesis in the cell. Our results show that *N-*glycosylation plays an important role in maintaining cell physiology as well as cellular morphology.

### Role of *N-*glycosylation in host-pathogen interaction.

**FIG 5:**
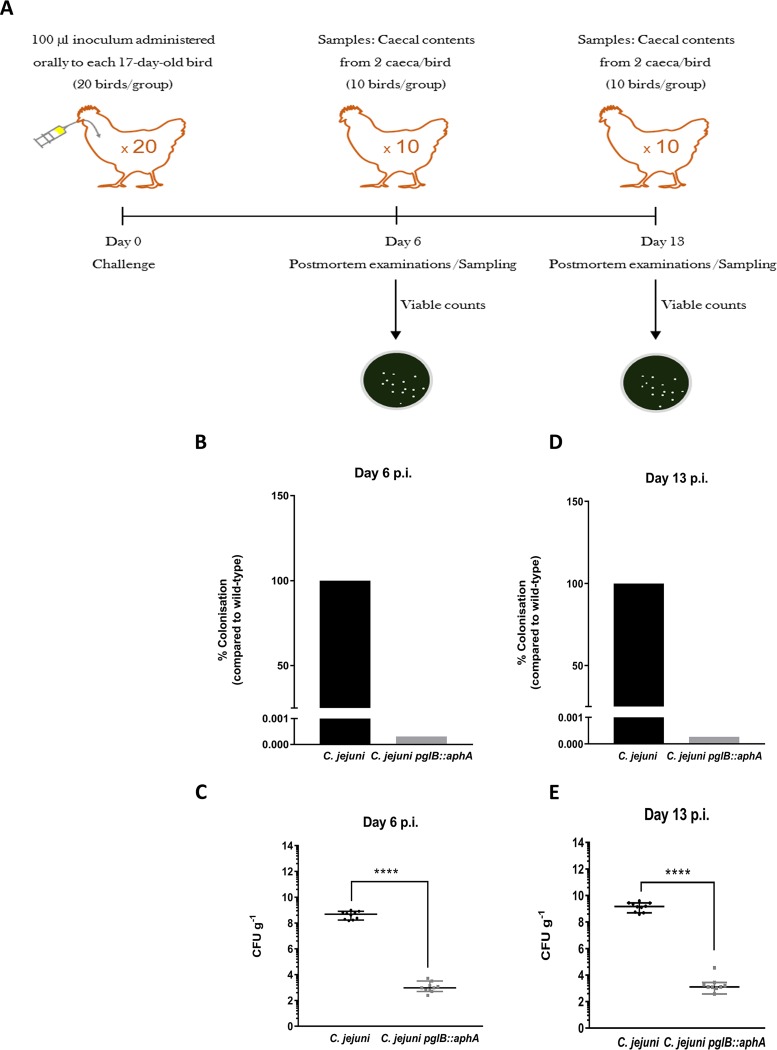
Colonization of C. jejuni and C. jejuni *pglB*::*aphA* in 17-day-old White Leghorn chickens. (A) Schematic diagram of the chicken colonization experiment. Seventeen-day-old chicken were inoculated with 100 μl of 10^6^ CFU of wild-type C. jejuni or C. jejuni *pglB*::*aphA*. Chickens were then sacrificed at day 6 and day 13, and postmortem examination (PM) was carried out by directly enumerating C. jejuni on CCDA plates. (B) CFU counts of C. jejuni strains on CCDA plates on day 6 postinoculation. (C) Percentage of C. jejuni strains colonization on day 6 postinoculation. (D) CFU counts of C. jejuni strains on CCDA plates on day 13 postinoculation. (E) Percentage of C. jejuni strain colonization on day 13 postinoculation. Statistical significance was calculated using Mann-Whitney test. ****, *P* < 0.0001.

To determine the impact of protein glycosylation in host-pathogen interaction, we conducted chicken colonization experiments. Owing to its prevalence in chicken and other avian species, many reports have used chicken models of infection to test the effects of mutations in C. jejuni (9, 34). We have previously reported that disruption of *pglH* substantially reduced colonization in 2-week-old Light Sussex chickens (9). The glycosyltransferase PglH catalyzes the addition of three α1,4- GalNAc to GalNAc-α1,4-GalNAc-α1,3-diBacNAc-β1, an important step in the production of the heptasaccharide. Proteins from C. jejuni *pglH*::*aphA* can still be glycosylated by the truncated heptasaccharide (GalNAc-α1,4-GalNAc-α1,3-diBacNAc-β1). To understand the role of glycosylation in chicken colonization, we sought to investigate the impact of a C. jejuni *pglB* mutant devoid of the ability to fully glycosylate proteins and compare it to the wild type. We separately inoculated groups of twenty 2-week-old *Campylobacter-*free White Leghorn chickens with 10^6^ CFU of wild-type strain of C. jejuni or the *pglB*::*aph* mutant (Fig. 5A). Ten chickens from each group were sacrificed at day 6 or 13 postinoculation, and viable C. jejuni in the ceca (a key site of persistence) were enumerated postmortem. Glycosylation-deficient C. jejuni *pglB*::*aph* showed a six-log_10_-unit reduction in cecal colonization compared to the wild type at day 6 (*P < *0.0001) and day 13 (*P < *0.0001) (Fig. 5B and C). This reduction in colonization is in agreement with our previous work on C. jejuni *pglH*::*aphA*. However, while glycan truncation in C. jejuni *pglH*::*aphA* reduced chicken colonization (9), the complete abolishment of glycosylation in C. jejuni *pglB*::*aphA* had a more severe impact on chicken colonization. The data highlight the importance of glycosylation in *Campylobacter* survival in the avian reservoir primarily responsible for zoonotic infections.

## DISCUSSION

Our results provide in-depth analyses on the alteration of the C. jejuni proteome as a consequence of disrupting the *N-*linked protein glycosylation pathway. Loss of glycosylation predominantly has a mass effect on the C. jejuni proteome, rather than being restricted exclusively to glyco-modified proteins. Previous studies have reported that loss of glycosylation affects bacterial motility and virulence (4, 35). However, none of these studies investigated the relative quantities of individual protein(s) and their function(s). Our TMT LC-MS/MS strategy shows that disruption of *N-*linked glycosylation correlates with a marked increase in the abundance of not only periplasmic but also cytoplasmic chaperones and proteases likely to be involved in folding, disaggregation, and degradation of proteins. These results suggest a critical defect in protein folding that potentially impairs protein cellular function. Differential abundance of protein quality control machineries was not translated into an obvious change in growth kinetics or cell dry weight. The cell count of the C. jejuni *pglB*::*aphA* strain at an OD_600_ of 0.4 to 0.5 showed a twofold reduction compared to wild-type C. jejuni; however, both strains had similar dry cell weights and growth kinetics (see Fig. S1 in the supplemental material). The reason(s) why the cell count was reduced at this OD_600_ in glycosylation-deficient C. jejuni strains remains unclear. One explanation may involve the observed reduction in the abundance of the cell division protein FtsZ (*P = *0.006) (Fig. 1A), suggesting major cellular alterations upon abolishing *N-*linked glycosylation. Functional analysis demonstrates that molecular assemblies and protein functions were statistically impaired in glycosylation-deficient C. jejuni. We postulate that the difference in extrusion kinetics of ethidium bromide in C. jejuni strains were dependent on the C. jejuni heptasaccharide. While the abundance of CmeABC was similar in wild-type C. jejuni and C. jejuni *pglB*::*aphA*, we observed a significant impairment in ethidium bromide extrusion in the glycosylation-deficient strain, even when compared to the C. jejuni *cmeB*::*aphA*. This might be due to a central role of glycans in stabilizing not only CmeABC as the major multidrug efflux pump but also CmeDEF as a secondary pump (36). Indeed, CmeE, a periplasmic fusion glycoprotein in C. jejuni (6), was significantly reduced in C. jejuni *pglB*::*aphA* (*P* < 0.0001). The low abundance of CmeE suggests that CmeDEF is inefficiently assembled, and therefore functionally impaired. By testing other molecular assembly functions, we noticed a general role played by the C. jejuni heptasaccharide. Notably, the high abundance in the protein level of NapAB in C. jejuni *pglB*::*aphA* was not accompanied by an increase in the nitrate reduction function of the complex, indicating a severe impairment in the function of NapAB resulting from the loss of *N-*linked glycans. The critical effects caused by *N-*glycosylation disruption are extended beyond cellular activities and the assembly of protein complexes. In C. jejuni, various oligosaccharides such as LOS and CPS and *N-*linked glycan pathways compete for the limited pool of Und-P. Disrupting *N-*glycosylation in C. jejuni (and other bacteria with general glycosylation systems) may lead to a secondary effect such as sequestering of Und-P so that it will not be readily available for other pathways such as peptidoglycan biosynthesis, leading to the cell morphology alterations observed in C. jejuni *pglB*::*aphA.* In similar studies, interruption of the biosynthesis of the O antigen in Escherichiacoli led to morphological abnormalities in cell shape (37). This phenotype was rescued by overexpressing UppS to increase the pool of Und-P (37). Scrutiny of our proteomic data shows a reduction in the abundance of UppS as well as MEP pathway enzymes in glycosylation-deficient C. jejuni. This suggests either a feedback loop mechanism that prevents the accumulation of Und-P intermediates, which might result in cell toxicity, or perhaps a biophysical role played by *N-*linked glycans that affects cell shape.

10.1128/mBio.00297-19.1 FIG S1 Bacterial cell count in Campylobacter jejuni at an OD_600_ of 0.4 to 0.5. (A) Cell enumeration at log 10^6^ of wild-type C. jejuni and C. jejuni *pglB*::*aphA*. (B) Dry cell weight of C. jejuni and C. jejuni *pglB*::*aphA.* Data show the means for three biological replicates with two technical replicates each, and error bars represent standard deviations. Significance was calculated using Student’s *t* test. ***, *P < *0.0005 Download FIG S1, PDF file, 0.3 MB. Copyright © 2019 Abouelhadid et al. 2019 Abouelhadid et al. This content is distributed under the terms of the Creative Commons Attribution 4.0 International license.

We performed reverse transcriptase PCR (RT-PCR) to rule out any polar effects resulting from disrupting *pglB*. Thus, transcription of the *pglA* and *pglC* genes downstream of *pglB* were similar in both wild-type and glycosylation-deficient C. jejuni (Fig. S2). Our results show a dramatic decrease in chicken colonization with C. jejuni *pglB*::*aphA* exhibiting a far greater decrease than that previously reported for C. jejuni *pglH*::*aphA* (9). This means that in contrast to the C. jejuni *pglH*::*aphA* mutant where a truncated glycan is produced, the full *N-*linked glycan is produced in the C. jejuni *pglB*::*aphA* strain but is not transferred to the acceptor proteins. This observation provides deeper insight into the essential role of the first two or three monosaccharides (GalNAc-α1,4-GalNAc-α1,3-diBacNAc) at the reducing end (which are absolutely conserved among strains in the *Campylobacter* genus) (7, 38) in host-microbe interactions. This study adds to understanding of the biological role of this general *N-*linked glycosylation system. We present a platform that enables the study of glycan-protein structural and functional relationship and could be applied to other prokaryotic protein glycosylation systems. Efforts to couple transcriptional and proteomic analysis to interrogate molecular changes resulting from altering or abolishing general glycosylation pathways in bacteria will enrich our understanding of the evolution and emergence of protein posttranslational modifications.

10.1128/mBio.00297-19.2 FIG S2 Organization of Campylobacter jejuni 11168H protein glycosylation locus. (A) Genes in the C.jejuni 11168H glycosylation locus indicated by arrows upstream and downstream of *pglB.* Arrow sizes are not to scale. Glycan biosynthesis genes (gray arrows), glycan translocation (light blue arrow), glycosyltransferases (dark blue arrows), oligosaccharyltransferases (black arrows), and the *aphA* gene from C. coli inserted to inactivate *pglB* in C. jejuni *pglB*::*aphA* strain (dark red rectangle) are indicated. (B) Transcription analysis of genes upstream and downstream to *pglB* in wild-type C. jejuni and C. jejuni *pglB*::*aphA.* Lanes 1, 5, and 9, RNA template from C. jejuni; lanes 3, 7, and 11, RNA template from C. jejuni *pglB*::*aphA*; lane 2, cDNA from C. jejuni probed by *pglJ* primers; lane 4, cDNA from C. jejuni *pglB*::*aphA* probed by *pglJ* primers; lane 6, cDNA from C. jejuni probed by *pglA* primers; lane 8, cDNA from C. jejuni *pglB*::*aphA* probed by *pglA* primers; lane 10, cDNA from C. jejuni *pglB* probed by *pglC* primers; lane 12, cDNA from C. jejuni *pglB*::*aphA* probed by *pglC* primers. Download FIG S2, TIF file, 0.2 MB. Copyright © 2019 Abouelhadid et al. 2019 Abouelhadid et al. This content is distributed under the terms of the Creative Commons Attribution 4.0 International license.

## MATERIALS AND METHODS

### Bacterial strains and growth conditions.

Campylobacter jejuni 11168H (39) and its derivatives, C. jejuni *pglB*::*aphA*, C. jejuni *cmeB*::*aphA*, and C. jejuni *napA*::*aphA*, were used in this study. C. jejuni 11168H was grown on Columbia-based agar or Mueller-Hinton-based agar supplemented with 5% horse blood according to the manufacturer’s instructions. *Campylobacter* strains were grown at 37°C in a variable atmospheric incubator (VAIN) cabinet (Don Whitley Scientific, UK) maintaining microaerophilic conditions of 85% nitrogen, 5% oxygen, and 10% carbon dioxide.

### Reverse transcriptase PCR.

Overnight cultures of wild-type C. jejuni and C. jejuni *pglB*::*aphA* were diluted to an OD_600_ of 0.1 and then incubated in the VAIN at 37°C under shaking conditions. At an OD_600_ of 0.4 to 0.5, cells were harvested, and RNA was extracted by using a Monarch total RNA extraction kit (New England Biolabs) following the manufacturer’s protocol. The extracted RNA (2 μg) was treated with Ambion Turbo DNase (Invitrogen) according to the manufacturer’s instructions. cDNA was generated using Superscript III kit (Invitrogen) from DNase-treated RNA. cDNA and DNase-treated RNA were used as the templates in PCR using MyTaq mastermix (Bioline, UK) using the *pglA, pglJ*, and *pglC* oligonucleotide primers in Table 1.

**TABLE 1:** Bacterial strains, plasmids, and oligonucleotide primers used in this study

Bacterial strain, plasmid, or primer	Description or sequence	Source or reference
E. coli strains		
DH10B	F^−^ *mcrA* Δ(*mrr*-*hsdRMS*-*mcrBC*) φ80d*lacZ*ΔM15 Δ*lacX74 endA1 recA1 deoR* Δ(*ara leu*)*7697 araD139 galU galK nupG rpsL* λ^−^	New England Biolabs, UK
*dam dcm* mutant	*ara-14 leuB6 fhuA31 lacY1 tsx78 glnV44 galK2 galT22 mcrA dcm-6 hisG4 rfbD1* R(*zgb210*::Tn*10*) Tet^s^ *endA1 rspL136* (Str^r^) *dam13*::Tn*9* (Cam^r^) *xylA-5 mtl-1* * thi-1 mcrB1 hsdR2*	New England Biolabs, UK
C. jejuni strains		
11168H *pglB::aphA*	C. jejuni 11168H *pglB* is inactivated by *aphA* encoding kanamycin resistance cassette	This study
11168H	Hypermotile variant of C. jejuni 11168	9
11168H *cmeB*::*aphA*	C. jejuni 11168H *cmeB* is inactivated by *aphA* encoding kanamycin resistance cassette	This study
11168H *napA*::*aphA*	C. jejuni 11168H *napA* is inactivated by *aphA* encoding kanamycin resistance cassette	This study

Plasmids		
pJMK30	*aphA* gene cloned in BamHI restriction site	41
pRSA	*cmeB* amplicon cloned in pJET1.2	This study
pRSF	*aphA* gene cloned in BamHI to inactivate *cmeB* in pRSA	This study
pATT3	*napA* cloned in pJET1.2	This study
pATT3F	*aphA* cloned in ClaI site inactivates *napA* in pATT3	This study

Oligonucleotides		
FWDcmeB	GACGTAATGAAGGAGAGCCA	
REVcmeB	CTGATCCACTCCAAGCTATG	
FWDnapA	ACCGCTATTGCAAGTGCTGCTAG	
REVnapA	GAAAGCGGACAAGTCGCATCC	
RTFWDPglA	ATG GGC AAA AAT TCC TTA TCG TTT	
RTREVPglA	GCT CGA CTA TAT CAC TTC TAG	
RTFWDPglC	CTC CGG TGA TTT TAA TCA CTG	
RTREVPglC	TTG CTT ACC CCA CTT CGT TT	
RTFWD PglJ	TTA GGA AGT GGT GGT GCT GA	
RTREV PglJ	AAA GCT ATG TCT TCA AGG GC	

### Protein preparation and digestion.

C. jejuni bacteria were grown overnight in brucella broth under microaerophilic conditions at 37°C under shaking conditions at 75 rpm. The following day, 3.5 ml was withdrawn to inoculate a 30-ml culture to a final OD_600_ of 0.1. The cultures were then incubated as described above until an OD_600_ of 0.4 to 0.5 was reached. Cells were harvested by centrifugation at 4,000 × *g* for 45 min. Supernatant was discarded, and cells were resuspended in lysis buffer (7 M urea, 2 M thiourea, 5 mM DTT, and 2% [wt/vol] CHAPS) and ultrasonicated following the manufacturer’s procedure for extraction of native protein samples from yeast cells (Covaris, US). Protein concentration was then measured using Bradford reagant (Sigma, UK) following the manufacturer’s protocol and then normalized to a total protein amount of 25 μg for enzymatic digestion. Prior to enzymatic digestion and labeling, samples were loaded into a stack gel for lysis buffer cleanup to eliminate any chemical interference at the labeling stage and to compress the whole proteome into a single band. Sample volumes were dried to half their original volume in a SpeedVac (Thermo Fisher Scientific, UK) with the volume replaced by Laemmli buffer (2×) and boiled for 10 min at 96°C. Reduced samples were loaded on to a 10% BisTris NuPAGE gel (Invitrogen, UK) and resolved for 10 min (100 V; 59 mA; 6 W) to “stack” the whole sample into a single band. Protein bands were visualized using Imperial protein stain (Thermo Fisher Scientific, UK). Gel bands were then excised, chopped, washed with acetonitrile (ACN), dried, and reduced by rehydration in 10 mM DTT for 30 min at 56°C. Gel slices were then washed with ACN, dried, and alkylated by incubation in 55 mM IAA at ambient temperature for 20 min in the dark, followed by washing with 100 mM TEAB, drying, and incubation in 40 μl trypsin (1.25 μg) at 37°C overnight. Peptides were extracted by collecting reaction supernatant.

### Peptide TMT labeling.

Each sample was treated individually with labels added at a 1:1 ratio. TMT6plex labels (Thermo Fisher Scientific, UK) were resuspended in 41 μl ACN, vortexed, and centrifuged for 1 min at 14,000 rpm. Each vial of TMT reagent was added to the appropriate sample, vortexed, and briefly centrifuged at 14,000 rpm before incubation at room temperature for 1 h. The reaction was stopped by adding 8 μl of 5% hydroxylamine (Sigma, UK) to each sample and incubated for a further 15 min at room temperature. Samples were then combined for each TMT6plex and incubated at room temperature for 15 min, followed by freezing at –80°C and drying in a SpeedVac.

### LC-MS/MS tandem mass spectrometry.

The combined TMT-labeled peptide sample was resuspended in a solution containing water-acetonitrile-trifluoroacetic acid (98%:2%:0.05%) and analyzed by LC-MS/MS. Chromatographic separations were performed using an Ultimate 3000 UHPLC system (Thermo Fisher Scientific, UK). A 3-μl injection of peptides (equivalent to 15 μg peptides) was resolved by reversed phase chromatography on a 75-μm C_18_ column (50 cm) using a three-step linear gradient of acetonitrile in 0.1% formic acid. The gradient was designed to elute the peptides at a flow rate of 250 nl/min over 120 min. The eluate was ionized by electrospray ionization using an Orbitrap Fusion Lumos mass spectrometer (Thermo Fisher Scientific, UK) and Xcalibur v4.1 software. The instrument was programmed to acquire data in the automated data-dependent switching mode, selecting precursor ions based on their intensity for sequencing by higher-energy C-trap dissociation (HCD) for peptide identification and reporter ion fragmentation. Selection of precursor ions was based on their intensity for sequencing by HCD in a TopN method. The MS/MS analyses were conducted using higher than normal collision energy profiles that were chosen based on the mass-to-charge ratio (*m/z*) and the charge state of the peptide. To increase fragmented peptide coverage and reporter ion intensities, a further synchronous precursor scan (SPS) of the top five most intense peaks using MS3 was performed.

Raw mass spectrometry data were processed into peak list files using Proteome Discoverer (Thermo Scientific; v2.2). The raw data file was processed and searched using the Mascot search algorithm (v2.6.0) (Matrix Science, Boston, MA, USA) and the Sequest search algorithm against the current Campylobacter jejuni 11168 proteome curated within Uniprot. Following processing with Proteome Discoverer, the resulting file was exported into Perseus (v1.6.1) (http://www.perseus-framework.org) for qualitative and quantitative data analysis.

### Construction of C. jejuni *pglB*::*aphA*, C. jejuni *cmeB*::*aphA*, and C. jejuni *napA*::*aphA*.

Construction of C. jejuni *pglB*::*aphA* was achieved by nonpolar insertion of the *aphA* gene into a unique restriction site present in the *pglB* gene of the pUC18 plasmid generated from a random genomic library used in sequencing C. jejuni 11168 (40). Briefly, pUC18 was digested using PsiI in the *pglB* gene, and linearized plasmid was then blunt ended using T4 DNA polymerase (Promega) according to the manufacturer’s protocol. The antibiotic resistance gene *aphA* was amplified and cut with StuI and blunt end ligated to the linearized plasmid. The construct was then electroporated in C. jejuni 11168H, and colonies were selected on CBA supplemented with kanamycin (30 μg/ml). Inactivation of *cmeB* in C. jejuni 11168H was achieved using the method described by Pumbwe and Piddock (26). Briefly, 1 kbp of *cmeB* was amplified with Phusion polymerase (New England Biolabs, UK) by primers FWDcmeB and REVcmeB using C. jejuni 11168H genomic DNA as the template. The amplicon was cloned into pJET1.2 to give pRSA. In order to disrupt the gene, the kanamycin resistance gene *aphA* was cloned in the middle of the *cmeB* fragment to give pRSF. Construction of C. jejuni 11168H *napA*::*aphA* was achieved by amplifying the *napA* gene with Phusion polymerase (New England Biolabs, UK) using primers FWDnapA and REVnapA using C. jejuni 11168H genomic DNA as the template. The amplicon was then ligated into pGEM T-easy to give pATT3, which was transformed to E. coli *dam dcm* mutant. To disrupt *napA*, *aphA* was cut with BamHI from pJWK30, blunt ended by CloneJET PCR cloning kit blunting enzyme (Thermo Fisher Scientific, UK), and then ligated to pATT3 that was previously cut by ClaI and blunt ended to give pATT3F. pRSF and pATT3F were electroporated in C. jejuni 11168H, and colonies were selected on CBA supplemented with kanamycin (30 μg/ml). All insertional inactivation were checked by gene-specific and *aphA*-specific primers.

### Antibiotic sensitivity.

C. jejuni 11168H was grown in suspension in Mueller-Hinton broth equivalent to a 1.0 McFarland standard, and 100-μl aliquots were spread plated on dry Mueller-Hinton agar plates supplemented with 5% sheep blood (Oxoid, UK). The plates were left for 5 to 10 min to dry before the antibiotic strip (Oxoid, UK) was added. The plates were incubated at 37°C overnight. The MIC was read directly from the strip at the point where the zone of inhibition of bacterial growth intersected with the antibiotic concentration on the strip.

### Ethidium bromide accumulation assay.

Bacterial cells were grown to mid-log phase (OD_600_ of 0.4 to 0.5). Cells were harvested, washed, and resuspended in 0.1 M sodium phosphate buffer (pH 7) (previously incubated in the VAIN) to an OD_600_ of 0.2. Cells were then incubated in the VAIN for 15 min at 37°C before a 100-μl aliquot was withdrawn to indicate time zero. Ethidium bromide (Sigma, UK) was added to a final concentration of 2 μg/ml, and fluorescence was measured at 530 nm excitation and 600 nm emission using a plate reader (Molecular Devices M3 plate reader, USA).

### Determination of the nitrite concentrations in the culture supernatants.

Determining the nitrite concentrations in the culture supernatants was done according to the method of Pittman et al. (25) with slight modifications. Briefly, an overnight culture of C. jejuni grown in brucella broth was diluted to an OD_600_ of 0.1 in oxygen-limited condition. Samples were drawn every hour and centrifuged at 12,000 × *g* for 1 min. Supernatant was then diluted 1:5 with deionized water. Fifty microliters of diluted culture supernatant was mixed with 850 μl of 1% (wt/vol) sulfanilamide dissolved in 1 M HCl and 100 μl of 0.02% (wt/vol) naphthylethylenediamine. After 15 min, the absorbance at 540 nm was measured using a plate reader (Molecular Devices M3 plate reader, USA), and nitrite concentrations were determined by reference to a standard curve.

### Scanning electron microscopy.

C. jejuni cells were resuspended in PBS after being scraped from an overnight culture grown on Columbia-based agar plate supplemented with blood and Skirrow supplement. The bacterial suspension was then mixed with fixing buffer (0.1 M sodium cacodylate buffer containing 4% paraformaldehyde [pH 7.4]) and fixed on polylysine-coated coverslips overnight at 4°C. Samples were then washed twice for 10 min in 0.15 M sodium cacodylate buffer (pH 7.3) and then treated for 1 h at room temperature with 1% osmium tetroxide in 0.15 M cacodylate phosphate buffer. Samples were dehydrated in an ethanol series consisting of the following steps: 10 min in 10% ethanol, 30 min in 70% ethanol, and three times in 100% ethanol (20 min each). The coverslips were then transferring to 100% ethanol in critical point drying holders and then critical point dried using carbon dioxide in a Polaron E3000 critical point dryer with >3 flushes over a 45-min soaking period. The coverslips were mounted on stubs (TAAB, UK) with conductive carbon adhesive discs (TAAB), and samples were sputter coated with gold in an Emitech K550 sputter coater (45 mA current, 2 min). The cells were viewed, and images were recorded using a FEI Quanta 200 field emission scanning electron microscope (FEI UK Ltd.) operated at 10 to 20 kV in high vacuum mode.

### Chicken colonization.

All procedures were conducted under Home Office project license PPL 60/4420, according to the requirements of the Animal (Scientific Procedures) Act 1986, with the approval of the Ethical Review Committee of the Moredun Research Institute. A total of 40 White Leghorn chickens, obtained on the day of hatch from a Home Office licensed breeding establishment were used. Chickens were housed in groups of 20 in colony cages under specific-pathogen-free conditions. Groups contained female and male chickens, and the wings of the chickens were tagged for individual identification. Water and sterile irradiated feed based on vegetable protein (DBM Ltd., UK) was provided *ad libitum*. Fresh fecal samples collected from the cages were plated onto mCCDA plates to confirm the absence of C. jejuni in the chickens. When the chickens were 2 weeks old, they were challenged with 10^6^ CFU of C. jejuni 11168H or 11168H *pglB*::*aphA* in a volume of 100 μl. At 1 and 2 weeks after challenge, postmortem examinations of 10 birds from each group were performed. The contents of each cecum from each bird were weighed and serially diluted in phosphate-buffered saline (PBS) and then plated on mCCDA plates to enumerate viable *Campylobacter* per gram of cecal contents. Statistical analysis was performed using the Mann-Whitney test in Graphpad Prism 7.

### Data availability.

The raw quantitative proteomic data have been deposited to the ProteomeXchange consortium (http://www.proteomexchange.org/) via the PRIDE partner repository with identifier PXD011452.

10.1128/mBio.00297-19.4 TABLE S2 Full list of proteins identified and compared in the wild-type C. jejuni and C. jejuni *pglB*::*aphA* strains in this study. Download Table S2, CSV file, 0.1 MB. Copyright © 2019 Abouelhadid et al. 2019 Abouelhadid et al. This content is distributed under the terms of the Creative Commons Attribution 4.0 International license.

10.1128/mBio.00297-19.5 TABLE S3 List of proteins found to show statistically different abundances in wild-type C. jejuni and C. jejuni *pglB*::*aphA.* Download Table S3, XLS file, 0.3 MB. Copyright © 2019 Abouelhadid et al. 2019 Abouelhadid et al. This content is distributed under the terms of the Creative Commons Attribution 4.0 International license.

## ADDENDUM

While this article was in process, an article on a similar topic was published in Molecular & Cellular Proteomics: Cain JA, et al. 2019. Proteomics reveals multiple phenotypes associated with N-linked glycosylation in Campylobacter jejuni. Mol Cell Proteomics 18:715–734. https://doi.org/10.1074/mcp.ra118.001199.
