# Supplementary figures and images for: Quantitative Analyses Reveal Novel Roles for N-Glycosylation in a Major Enteric Bacterial Pathogen
Source: mBio. 2019 Apr 23;10(2):e00297-19. doi: 10.1128/mBio.00297-19 (PMC6478998; doi:10.1128/mBio.00297-19)

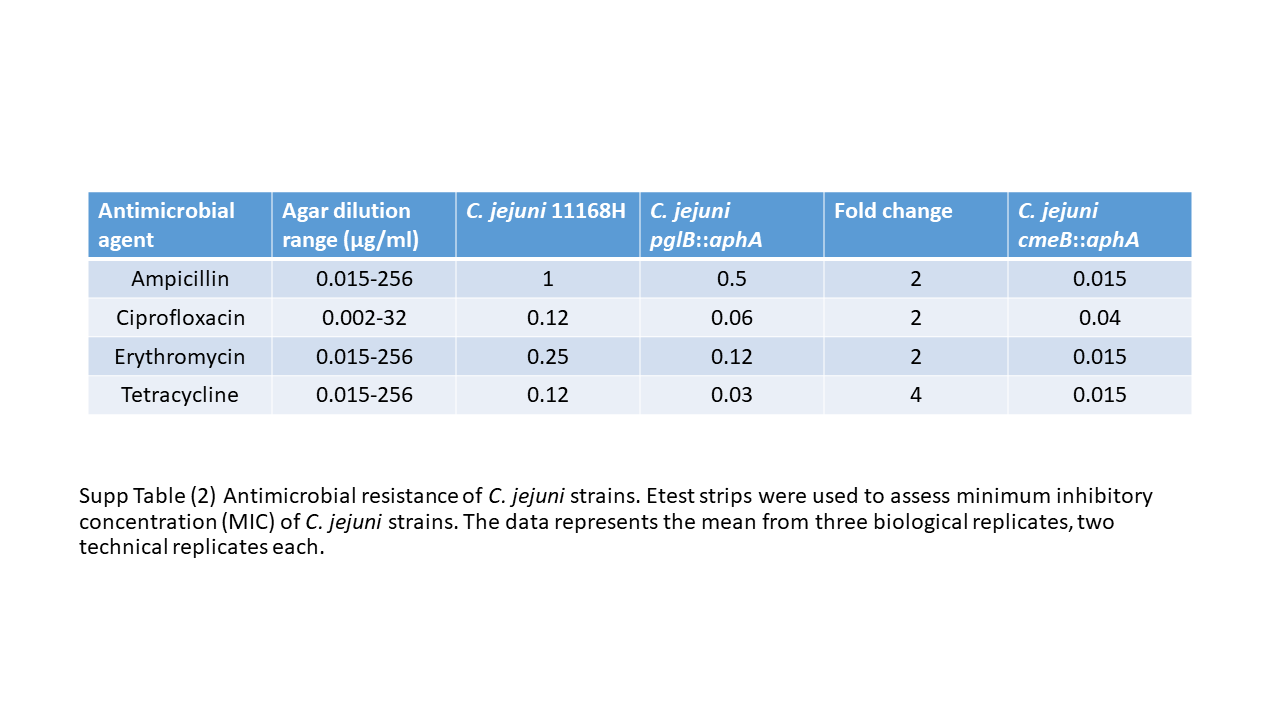

Supplement: TABLE S1 [file mBio.00297-19-st001.tif]

# CFU at O.D<sub>600</sub>= 0.4 - 0.5

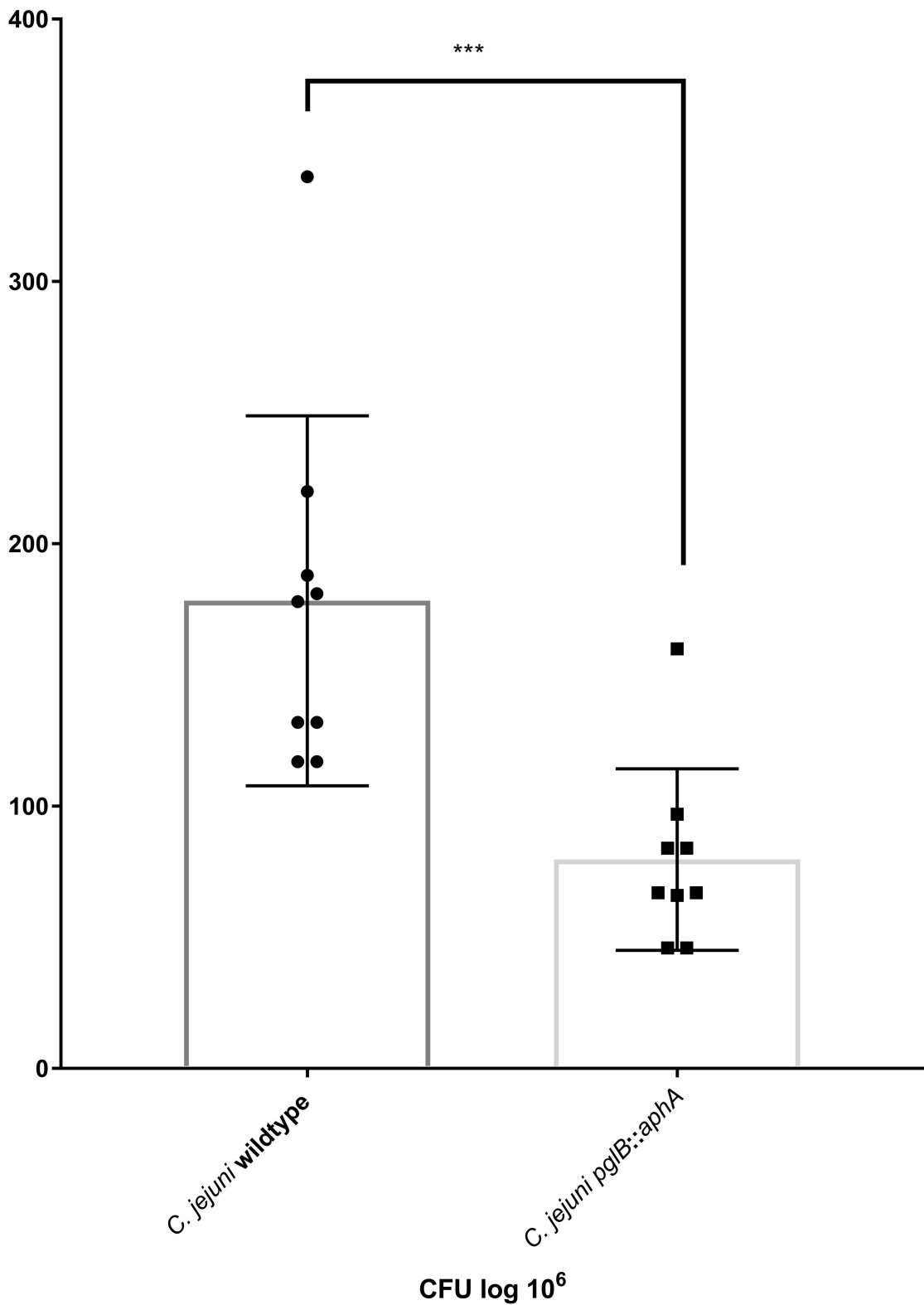

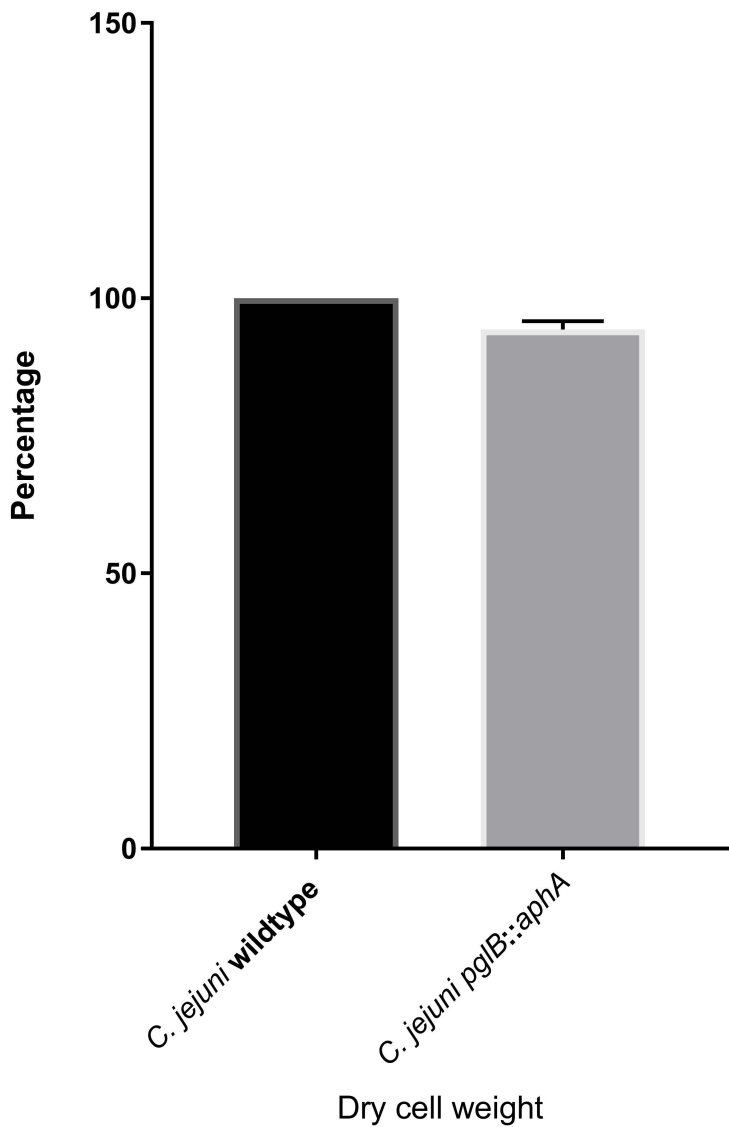

Supplement: FIG S1 [file mBio.00297-19-sf001.pdf]

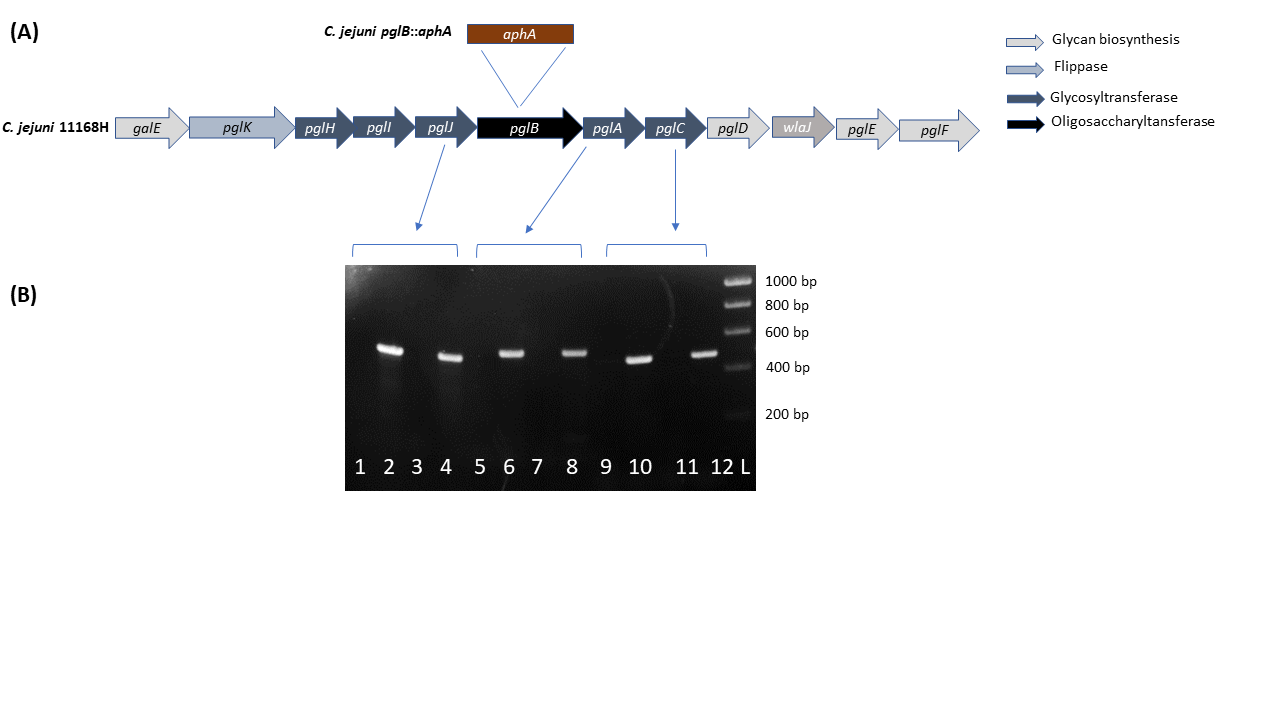

Supplement: FIG S2 [file mBio.00297-19-sf002.tif]
